# Cutting through the smoke: the diversity of microorganisms in deep-sea hydrothermal plumes

**DOI:** 10.1098/rsos.160829

**Published:** 2017-04-12

**Authors:** Anni Djurhuus, Svein-Ole Mikalsen, Helge-Ansgar Giebel, Alex D. Rogers

**Affiliations:** 1 Department of Zoology, University of Oxford, South Parks Road, OX1 3PS UK; 2 Department of Science and Technology, University of the Faroe Islands, Noatun 3, Torshavn, Faroe Islands; 3 Institute of Chemistry and Biology of the Marine Environment (ICBM), Carl-von-Ossietzky University Oldenburg, , Germany

**Keywords:** hydrothermal vents, microbial ecology, East Scotia Ridge, Southwest Indian Ridge

## Abstract

There are still notable gaps regarding the detailed distribution of microorganisms between and within insular habitats such as deep-sea hydrothermal vents. This study investigates the community composition of black smoker vent microorganisms in the Southern Hemisphere, and changes thereof along a spatial and chemical gradient ranging from the vent plume to surrounding waters. We sampled two hydrothermal vent fields, one at the South West Indian Ridge (SWIR), the other at the East Scotia Ridge (ESR). Samples were collected across vent fields at varying vertical distances from the origin of the plumes. The microbial data were sequenced on an Illumina MiSeq platform for the 16SrRNA gene. A substantial amount of vent-specific putative chemosynthetic microorganisms were found, particularly in samples from focused hydrothermal venting. Common vent-specific organisms from both vent fields were the genera *Arcobacter*, *Caminibacter* and *Sulfurimonas* from the Epsilonproteobacteria and the SUP05 group from the Gammaproteobacteria. There were no major differences in microbial composition between SWIR and ESR for focused plume samples. However, within the ESR the diffuse flow and focused samples differed significantly in microbial community composition and relative abundance. For Epsilonproteobacteria, we found evidence of niche-specificity to hydrothermal vent environments. This taxon decreased in abundance by three orders of magnitude from the vent orifice to background water. Epsilonproteobacteria distribution followed a distance–decay relationship as vent-effluents mixed with the surrounding seawater. This study demonstrates strong habitat affinity of vent microorganisms on a metre scale with distinct environmental selection.

## 1. Introduction

Hydrothermal vents are ubiquitous along mid-ocean ridges, back-arc basins and volcanically active seamounts. The fairly recent discovery of hydrothermal vents [1] has transformed our view of microbial ecology in the oceans. The microorganisms resident around hydrothermal vents, often chemosynthetic, supply the base of the food web for the numerous animals associated with these ecosystems. With a rich variety of environmental conditions and steep physical and chemical gradients [2], hydrothermal vent systems offer a range of habitats, which allow several microbial niches to be realized.

Recently, the phylogenetic and functional diversity of non-symbiotic microorganisms around hydrothermal vents is starting to be explored [3–5]. Chemolithotrophic organisms face the challenge of extracting energy from narrow redox zones in marine environments. Thus, non-symbiotic chemolithotrophic microorganisms typically occur in biofilms on sulfidic rocks or in filamentous mats, e.g. *Beggiatoa* spp., absorbing reduced compounds from the substrata below and oxygen from the water above. Contrarily, other non-symbiotic vent microorganisms namely the free-living planktonic microorganisms dwell in the unpredictable fluid environment around the vents, with unstable physico-chemical conditions and immense variation in temperature and availability of compounds. These conditions offer a variety of microhabitats that potentially could be occupied by diverse microorganisms. Several studies have documented the remarkable diversity of free-living planktonic microorganisms inhabiting the ambient water of vent discharge [6–9], with evidence that vents with different chemical characteristics harbour distinct microbial populations [5,10–12]. However, data are scarce on the roles and mechanisms of dispersion and nutrition [4,13,14]. Microbial taxa with the metabolic flexibility of rapid adaptation to changing geochemical conditions have an advantage around high-temperature plumes [15]. The Epsilon- and Gammaproteobacteria are found in almost all habitats surrounding hydrothermal vents [14,16–20]. The Epsilonproteobacteria are known to have diverse metabolic properties but are commonly found to be meso- or thermophilic hydrogen-oxidizing and sulfur-reducing chemolithotrophs [15]. Because of their preferences and high abundance in hydrothermal vent systems, Epsilonproteobacteria serve as an ideal taxon for examining vent-specific microbial community structures [7]. In addition to chemosynthetic microorganisms, Sheik *et al.* [5] demonstrated that ubiquitous water-column microorganisms also populate plume communities.

Multiple studies have explored the effects of dispersal limitation on microbial biogeography [21]. Biogeographic signals have been found for hyperthermophilic archaea, such as the deep-sea hydrothermal vent euryarchaeota, and sulfate-reducing bacteria in similar environments [22–24]. One detailed biogeographic study has been conducted on the hyperthermophilic chemoautotrophic genus *Persephonella* (phylum Aquificae) [25,26]. Interestingly, this study revealed a distinct biogeographic pattern in a relatively small area of the Pacific, even at the genus level. This suggests that considerable dispersal barriers exist for the migration of microorganisms to spatially distinct habitats. Collectively, these studies have shown that, as with metazoans, the genetic similarity of microorganisms is negatively correlated with distance, i.e. demonstrating a distance–decay relationship [22,25]. Therefore, we can presume that hydrothermal plumes with distinct chemical characteristics that are geographically separated would host different microbial communities. However, there are still gaps in our knowledge on microorganisms within plumes and their distribution patterns along chemical gradients and mechanisms thereof.

Investigations are rare of free-living microorganisms in hydrothermal plumes, especially in the Southern Hemisphere. Changes in microbial communities as vent effluents are diluted with ambient water as they emerge has not previously been studied. We report a study comparing free-living microorganisms from two Southern Hemisphere deep-sea hydrothermal vents at the South West Indian Ridge (SWIR) and the East Scotia Ridge (ESR). We investigate the spatial pattern of microbial community composition between these two vent fields and, by strategic sampling, show how the microbial community structure changes on a small scale (metres) across the ESR vent plumes. We hypothesize the microbial communities to not be the same between the ESR and SWIR based on geographical distance and differences in chemistry. We also expect the hydrothermal plumes to affect the local environment stimulating vent-specific microbial communities and show a distance–decay relationship with shifting chemical conditions.

## 2. Material and methods

### 2.1. Field site

**Figure 1.**
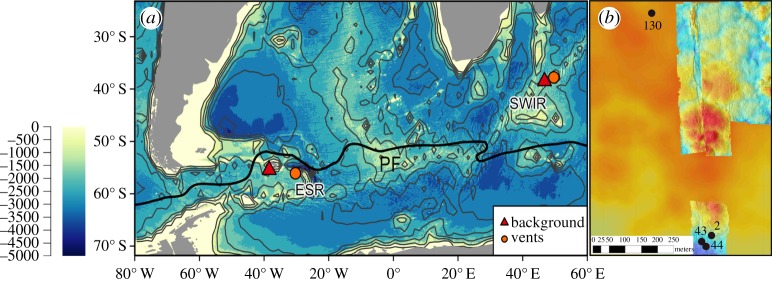
(*a*) Map showing the locations of samples in the Southern Ocean (ESR) and Indian Ocean (SWIR). PF: Polar Front. (*b*) The topography, indicated by colour, of the ESR vents at the E2 segment. The two focused venting stations from the ESR (Dog’s Head) are stations marked 43 and 44, the two diffuse vent stations are 2 and 130.

We conducted a comparative study of two vent sites in the Southern Hemisphere: the E2 site (30°19.1′ W, 56°05.3′ S) on the ESR and the Longqi vent field (49°38.942′ E, 37°47.022′ S) on the SWIR (figure 1).

The E2 segment, discovered in 2009, has been visited by several research cruises [8,27–32]. E2 is slow spreading approximately at 65–70 mm yr^−1^, but because of the large magma chamber underneath the vent field, it has similar characteristics to a faster spreading ridge [33,34]. The investigated vent site at E2 named ‘Dog’s Head’, consists of a complex of four chimneys up to approximately 12 m high that actively vent black smoker fluids at temperatures of up to 351°C [32]. The chemical composition of fluids from Dog’s Head consist of chloride (Cl^−^) concentrations similar to that of seawater and, consequently, have higher concentrations of the major cations, including sodium, and lower concentrations of volatiles such as hydrogen sulfide (H_2_S), [8,9,27,28,34–36].

The Longqi vent field, discovered in 2007, is the first active seepage found at an ultra-slow spreading ridge (less than 16 mm yr^−1^) [37–39]. This vent field is mostly constructed of chalcopyrite (CuFeS_2_), kusachiite (CuBi_2_O_4_), pyrite (FeS_2_), manganese phosphate hydrate and remarkably high concentrations of sulfate have been documented in some areas [37,39].

### 2.2. Sample collection

Sampling was carried out during Royal Research Ship *James Cook* voyages JC67 and JC80 during austral summer from 4 November to 21 December 2011 and 1 to 30 December 2012, respectively (figure 1*a*). For sample collection, we deployed a Seabird +911 conductivity, temperature and depth (CTD) profiler mounted on a titanium frame holding 24 externally sprung 10 l niskin bottles. The profiler was equipped with a light-scattering sensor (LSS) and a bespoke Eh electrode (Ko-ichi Nakamura) for detecting hydrothermal anomalies and reduced chemical species. The LSS and Eh sensors were used to estimate when the CTD was within hydrothermally influenced water. At E2, we used the Eh sensor as a measure of relative degree of mixing for hydrothermally influenced water with a lower Eh signifying a higher concentration of reducing chemical species implying closer to the origin of the plume. From Longqi, we had much fewer samples and did not have an Eh sensor for directly comparative work to ESR. We used the data from Longqi to compare total community structures between two vent systems.

**Figure 2.**
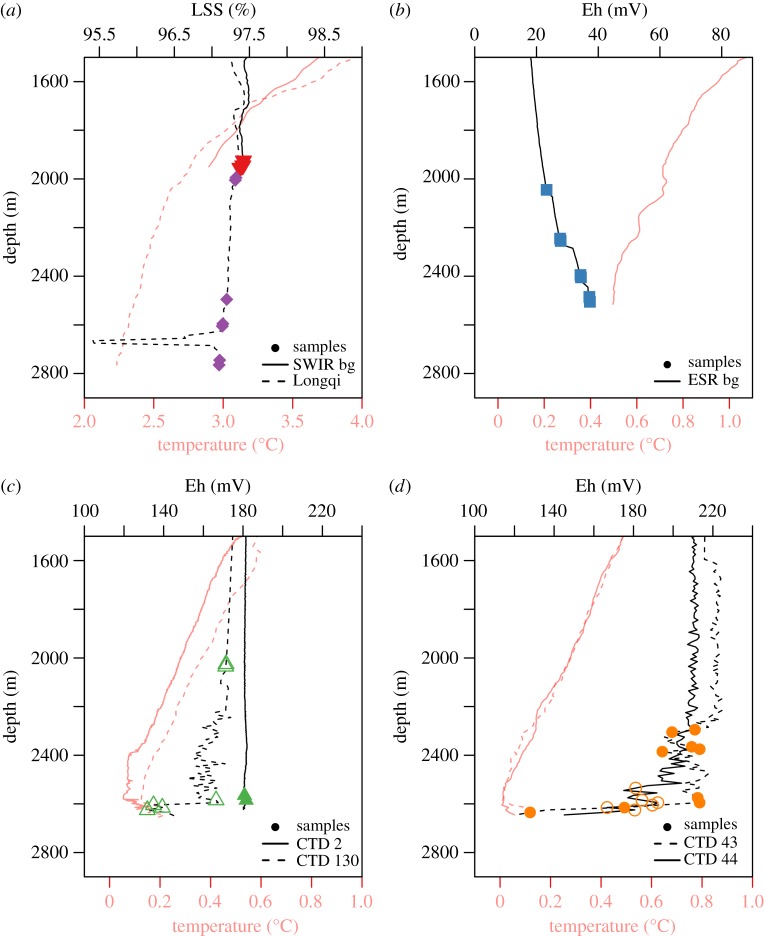
Eh potential, light scattering sensor (LSS) and temperature from CTD casts from the SWIR and the ESR. (*a*) The concentrations of LSS and temperature at the Longqi vent and background. Panels (*b*,*c*) and (*d*) show the temperature and Eh potential at the background, diffuse vents (cast 2 and 130) and Dog’s Head (cast 43 and 44), respectively. The symbols represent the location for each sample collected.

The samples collected around Dog’s Head (ESR) originated from high-temperature fluids, buoyant hydrothermal plumes and neutrally buoyant plumes. For comparison, we collected samples from diffuse areas of (low-temperature) venting and background seawater at E2. We collected samples from four different CTD casts: two were the same vent location designated as Dog’s Head (cast 43 and 44, figure 2) and the other two were from different diffuse vents located close to Dog’s Head but without any visually confirmed focused venting (casts 2 and 130, figure 2). At Longqi (SWIR), we collected samples from a single vertical CTD cast through the plume of a high-temperature vent (table 1) and a single background location.

**Table 1.** Sample overview. bg, background samples; DV, diffuse flow; depth, seafloor depth.

station	CTD cast	latitude	longitude	cells/ml	depth (m)	no. samples
bg ESR	1	55°26′66′′ S	38°25′63′′ W	24 875	2500	12
bg SWIR	11	38°31′56′′ S	46°45′74′′ E	147 786	1950	8
Dog’s Head	43, 44	56°05′35′′ S	30°19′13′′ W	16 581	2607	34
Longqi	18	49°38′94′′ S	37°47′02′′ E	98 959	2750	12
DV ESR	2, 130	56°04′62′′ S	30°19.41′ W	25 122	2610	30

The plume from Dog’s Head rises to 350–400 m above the seafloor [29], thus all samples collected at E2 deeper than 2000 m were considered vent-influenced samples. To ensure collection of non-vent microorganisms, we collected additional samples approximately 600 m from the seabed. Fluid samples collected over the hydrothermal vents were taken according to water column anomalies from the Eh detector on the CTD (figure 2). At each vent field, samples were collected along observed Eh (ESR), or LSS (SWIR) gradients to investigate microbial community structures relative to distance from plume. High Eh is correlated with distance from origins of the plume and is thus used as a proxy for plume dilution [40,41].

At each sampling point, duplicates of 1 l seawater were collected for microbial community structure analysis. All samples were collected on 0.2 μm pore size filters (polycarbonate membranes, 4.5 cm diameter, Sigma-Aldrich). All sampling was performed in a controlled temperature room at 4°C.

### 2.3. Flow cytometry analysis of bacterioplankton

Subsamples for flow cytometry were taken directly from the Niskin bottles, fixed with glutaraldehyde (1.0% final concentration), incubated for 15 min at 4°C and finally stored at −80°C until further analysis. Bacterial cell numbers were determined using an Accuri C6 flow cytometer (BD Biosciences, Franklin Lakes, NJ, USA) by SybrGreen I (SGI, Invitrogen, Carlsbad, CA, USA) staining. 1 μm Multicolour latex beads (Polysciences Europe, Eppelheim, Germany) were used as an internal standard. After 30 min of incubation in the dark, each sample was analysed using a flow rate of 14 μl min^−1^. Bacteria were detected using manual gating after visual inspection of the dot plots of the green versus red fluorescence and forward versus sideward scatters (FSC and SSC) and their histogram plots, respectively. The internal fluidics calibration of the device was used for volume calibration and verified by TruCount beads (BD Biosciences, Franklin Lakes, NJ, USA) as described previously [42]. Data were processed by BD Accuri C6 C-Flow software (version 1.026421).

### 2.4. Sequencing and data preparation

DNA extraction, PCR and sequencing were performed using a modified version of the Earth Microbiome Project (EMP) protocol adapted for the Illumina MiSeq [43]. In brief, the genomic DNA was extracted from subsamples of the filters using a Powersoil-htp 96 well DNA isolation kit (MoBio, Carlsbad, CA, USA) with a 10 min (65°C) incubation step modification. The V4–V5 region of the 16S rRNA gene was amplified with the 515F/806R primers with 12 base pair (bp) barcodes. The amplification primers were adapted from Caporaso *et al.* [44] to include nine extra bases in the adapter region of the forward amplification primer that support paired-end sequencing on the MiSeq. Amplifications were done in triplicates and followed the EMP PCR protocol. PCR products were pooled at equimolar concentrations and cleaned using the UltraClean^®^ PCR Clean-Up Kit (MoBio). 16S rRNA amplicon sequencing was conducted at the IGSB Next Generation Sequencing Core at Argonne National Laboratory (Chicago, USA), using 151 bp paired-end sequencing on an Illumina MiSeq instrument.

Quality filtering of reads was applied, as described previously [44]. Reads shorter than 75 bases and reads whose barcode did not match with an expected barcode were discarded. Forward and reverse raw sequences were combined and chimeras were removed. Joined reads were demultiplexed and quality trimmed using QIIME 1.8 [44]. An open-reference OTU (Operational Taxonomic Units) picking strategy was used, where OTUs were clustered against the GreenGenes 13_8 reference sequences using uclust [45] and reads with no hit to the reference sequence collection were subsequently clustered *de novo* at the 97% similarity level using uclust [45]. Reads were assigned to OTUs based on their best match to this database at greater than or equal to 97% sequence identity. PyNAST [43] was used to align OTU sequences and OTU taxonomy was assessed using the RDP classifier retrained towards the GreenGenes database (97% similarity) [46]. Median sequence counts per sample after OTU picking were 22 522 (s.d. 8321). To generate a final OTU table, sequences not aligning in the PyNAST step were removed, and a subsampled OTU table was created by random sampling to an even depth of 11 270 sequences per sample prior to any further analysis.

Taxonomy was assigned to each read by accepting the Greengenes taxonomy string of the best matching Greengenes sequence. All bioinformatics were conducted using QIIME [44].

### 2.5. Data analyses

Species richness was estimated from observed richness. Differences in bacterial cell abundances and richness between casts were calculated using an ANOVA and *post hoc* Tukey HSD tests.

A non-metric multidimensional scaling (NMDS) plot was conducted to understand the clustering of the microbial communities; a permutation test (10 000 permutations) was performed to check the significance of the NMDS model. For all ordination and richness analyses, the R package vegan was used [47]. *Post hoc* analyses between the locations (background ESR, background SWIR, Dog’s Head, ESR diffuse vents and Longqi) were performed with a permutational multivariate analysis of variance (PERMANOVA) using distance matrices with the function *adonis* from the R vegan package [48]. For visualization purposes only the taxa present in at least 20% of the samples were represented; however, all OTUs were included in statistical analysis. Recalculation of the raw sequence data was done using the R package phyloseq [49].

We calculated a divisive hierarchical clustering of all 80 samples using the Unweighted Pair Group Method with Arithmetic Mean (UPGMA) method using the cluster, and ape packages [49–51]. The relationship between sequence abundance of Epsilonproteobacteria and Eh redox potential was investigated using ordinary least-square linear regression. CTD data were analysed with the R package oce [52]. All data were analysed using the statistical software R v. 3.0.1 (R Core team 2015).

## 3. Results

### 3.1. Vent setting and abundance

The environmental setting at the SWIR and ESR vents reflects their respective positions north and south of the polar front based on temperature (figures 1 and 2) [53]. The background samples from both ridges show no hydrothermal vent signal and a stable declining temperature throughout the deep water column (figure 2). The LSS from the SWIR (Longqi) vent evidently show anomalies when approaching the hydrothermal vent. The two CTD casts above the ESR diffuse vents show clear Eh signals, more prominently from cast 2 than 130 (figure 2), but not as strong as for Dog’s Head (ESR). The two different casts from Dog’s Head are very similar (figure 2). The Eh potential suggests higher concentration of reducing chemical species at the vent sites (Dog’s Head) compared with the diffuse vents and background at the ESR, based on Eh potential.

**Figure 3.**
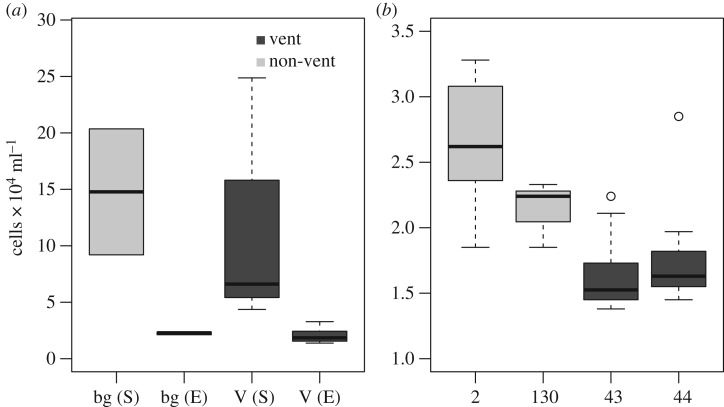
The boxplots show the abundance of microorganisms between the Longqi and Dog’s Head vent fields and their respective background samples (*a*) and between the four CTD casts collected from the ESR (V (E)) representing diffuse venting (2, 130) and black smoker venting (43, 44) (*b*). S is the SWIR and E is the ESR. V is vent or diffuse flow and bg is background. The figures have different scales on the *y*-axes.

Microbial abundances, as determined by flow cytometry, at the SWIR vent field (Longqi) averaged 98 959 (s.d. 68 649) cells ml^−1^ and 147 786 (s.d. 55 791) in the background. At a lower abundance, the ESR vent samples averaged 20 496 (s.d. 5577) cells ml^−1^ and 24 875 (s.d. 4904) cells ml^−1^ in the ESR background (figure 3). Bacterial abundances were not significantly different between the background and vent samples at the SWIR (Tukey HSD, *p*<0.09) and the ESR (Tukey HSD, *p*<0.99).

From the four ESR casts the community structure can be divided into two, the diffuse vents (cast 2 and 130) and the black smoker vents (casts 43 and 44, both at Dog’s Head), referred to as diffuse vents and Dog’s Head. The cell abundance from the diffuse vents showed a much higher abundance than the Dog’s Head samples with cast 43 significantly different from cast 2 (Tukey HSD *p*-value <0.01) and cast 130 (Tukey HSD *p*-value=0.0245) significantly different from cast 44 (Tukey HSD *p*<0.01; figure 3*a*).

### 3.2. 16S rRNA gene-based assessment of microbial community structures

After filtering and quality checking, we had a total of 2 million sequences from the 80 samples averaging at 22 522 (s.d. 8321) sequences per sample. In total, 25 700 taxa were determined based on a 97% similarity threshold. The total number of taxa for the ESR and SWIR backgrounds was 4970 and 5000, respectively. The number of taxa from the vent-specific samples were 7190 for the SWIR and 18 650 for the ESR, although these values are skewed towards locations with a greater sampling effort (i.e. vents at the ESR; electronic supplementary material, figure S1). The mean observed richness by location showed similar richness across the locations with a slightly lower variance of richness in the Dog’s Head (ESR) vent samples; however, based on an ANOVA, these patterns were not significant (*p*=0.366, *F*=0.824).

**Figure 4.**
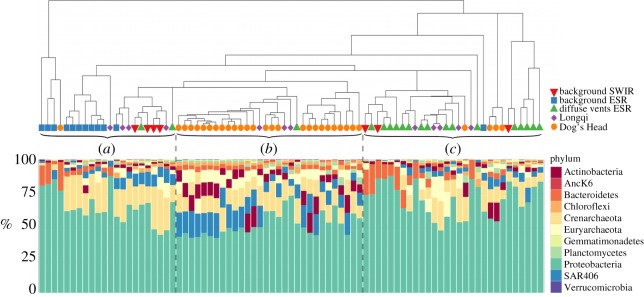
Hierarchical clustering dendrogram of free-living microorganisms. We designate the dendrogram into three groups, which represent background samples (*a*), vent samples (*b*) and diffuse vent samples (*c*). Barplots for each sample show the community composition of the most abundant phyla. For clarity only OTUs that were observed in more than 20% of the samples are shown.

**Figure 5.**
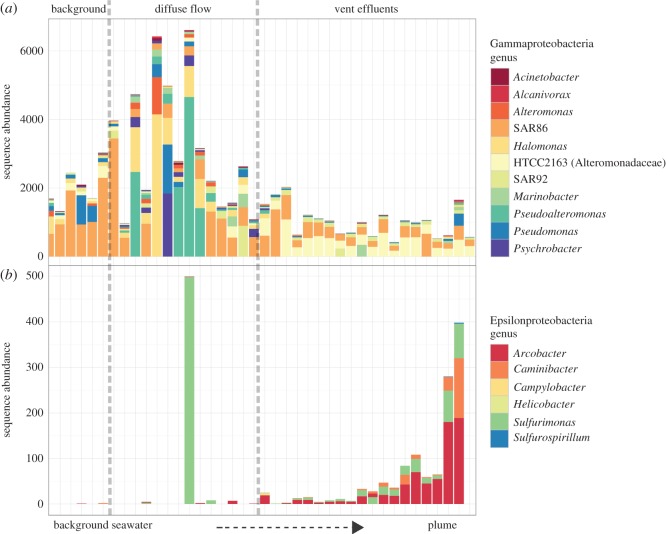
Barplot of Gamma- (*a*) and Epsilonproteobacteria (*b*) sequence abundances. From left to right increasing in Eh potential (indicative of plume signal) from background to diffuse vent to black smoker vents from the ESR. For clarity, only OTUs that were observed in more than 20% of the samples are shown.

The RDP classifier assigned most of the 16S rRNA sequences to Bacteria with only 14% classified as Archaea (figure 3). Proteobacteria was the most dominant phylum in all samples (58%); other abundant phyla include the Bacteroidetes (7.1%), Marine Group A (6.9%) [54] and Actinobacteria (5.7%, figure 4). Gamma- and Alphaproteobacteria represent the most abundant classes of the Proteobacteria of 53% and 24%, respectively, followed by Delta- (20%), Epsilon- (1.3%) and Betaproteobacteria (1.1%). While many taxa detected in all samples are common deep-sea microbes, the Epsilon- and Gammaproteobacteria, indicative of microaerobic reducing environments in the water column, suggest that samples containing those taxa were specifically collected from vent-influenced water. Half (49%) of the Gammaproteobacteria and Epsilonproteobacteria sequences were classified to genus level; and of the Gammaproteobacteria sequences the SAR92, SAR86, *Halomonas* and *Pseudoalteromonas* represented 75% of the sequences (figure 5*a*). Among the samples containing Epsilonproteobacteria the genera *Arcobacter*, *Sulfurimonas* and *Caminibacter* were clearly dominant. These three genera represent 99% of sequences assigned to genus level (figure 5*b*).

#### 3.2.1. Community differences between ESR and SWIR

Background samples from the ESR and SWIR contained a composition of phyla similar to the vent samples but with fewer Marine Group A and Actinobacteria and relatively more Proteobacteria (figure 4). At the class level, the ESR showed a relatively higher abundance of Betaproteobacteria and neither background cast had any Epsilonproteobacteria (electronic supplementary material, figure S2). However, these two background samples show differing patterns in the Gammaproteobacteria genera, with ESR background being completely dominated by the common marine taxon SAR86 at substantially higher relative abundances than the background of the SWIR. The background at the SWIR was also mostly dominated by SAR86, but also had high relative abundances of *Halomonas*. One sample, in particular, had a high relative abundance of genera *Halomonas* and *Alteromonas*, which resembles some of the diffuse vent samples from the ESR.

**Figure 6.**
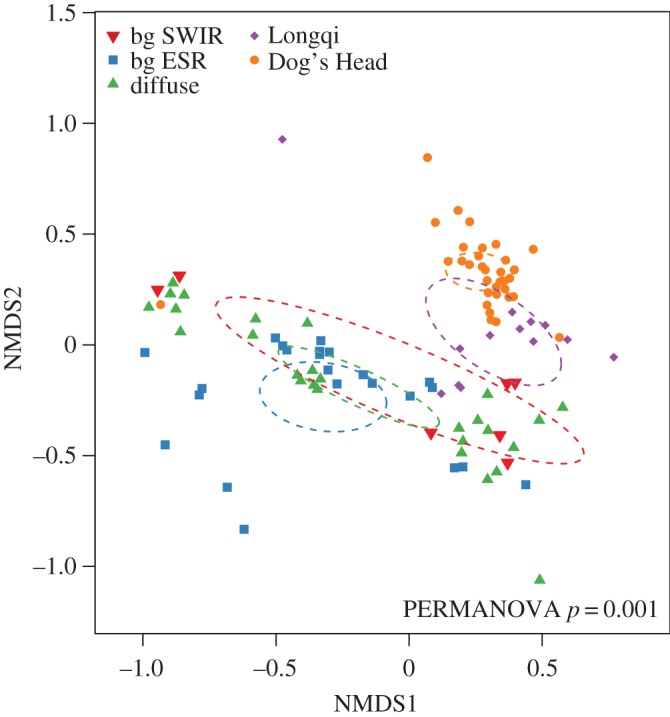
The non-metric multidimensional scaling (NMDS) of bacteria/microorganisms in all locations with their 95% standard error (ellipses).

The hierarchical clustering largely divided the microbial communities into background samples, ESR diffuse vents, and the focused vent samples (Longqi and Dog’s Head). This is further verified by the NMDS (figure 6), where the vent samples mostly clustered together and the diffuse samples and background samples from the SWIR and ESR clustered together.

#### 3.2.2. Dilution of microbial communities

Microbial composition between Dog’s Head and the E2 diffuse vents were distinct in their composition (figures 5 and 6). At the diffuse vent, Epsilonproteobacteria were barely present and *Psychrobacter* and *Pseudomonas* (Gammaproteobacteria) completely dominated the community. By contrast, the Gammaproteobacteria SAR92 and Epsilonproteobacteria dominate at Dog’s Head. From the Gammaproteobacteria, the sulfur-oxidizing family SUP05 was present in all vent and diffuse samples, but not found in the background samples (electronic supplementary material, figure S2).

**Figure 7.**
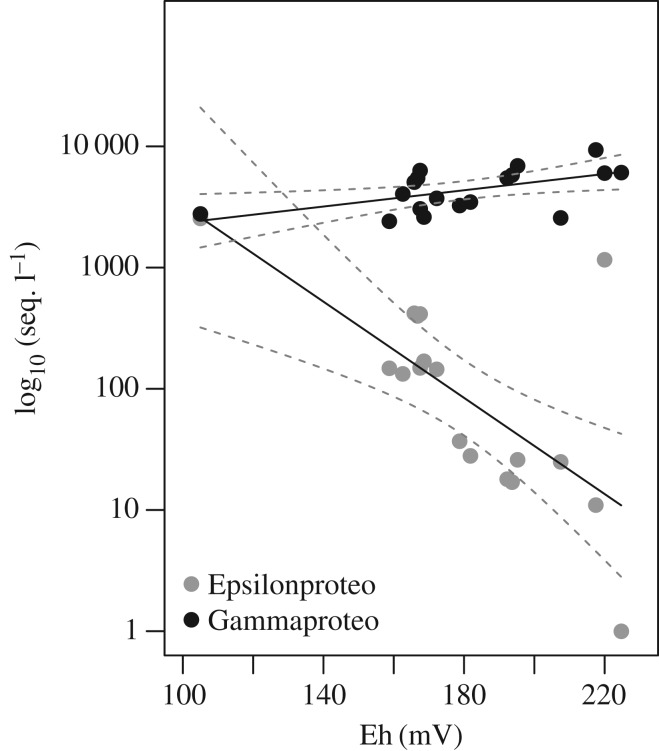
Linear model of sequence abundance of Gammaproteobacteria and Epsilonproteobacteria regressed against Eh potential from the focused vent sites from ESR.

The Epsilonproteobacteria from ESR show a declining sequence abundance with distance from vent, indicating dilution of the community with the background community. Epsilonproteobacteria sequence abundance shows a log-linear decline to increasing Eh (Pearson corr. test *r*^2^=0.77, *p*<0.01; figure 7), indicative of distance from active venting. Contrary to the Epsilonproteobacteria the Gammaproteobacteria have a positive correlation with distance from vent with sequence abundance (Pearson corr. test *r*^2^=0.25, *p*=0.019; figure 7). In addition, the Gammaproteobacteria show an increase in sequence abundance flourishing at the diffuse vents with distinct taxa, such as *Pseudoalteromonas*, *Halomonas* and *Psychrobacter*. The Gamma- and Epsilonproteobacteria show a distinct difference between Dog’s Head and the background community. A similar correlation was not attempted with LSS and the microorganisms from Longqi since the small sample size precludes us from a robust statistical inference.

## 4. Discussion

### 4.1. Environmental impact on vent microbial communities

Hydrothermal fluids are the product of chemical exchange between seawater and the ocean lithosphere at high temperatures in the deepest part of the hydrothermal convection cell [39,55]. Our results strongly indicate that the microbial community compositions at our sampling locations were related to the presence of reducing chemical species in the environment in line with previous research on the drivers of microbial assemblages and dispersion close to vent locations [3,7,37,39]. The taxa of the microbial assemblages described in this study are well documented at hydrothermal vent and other deep-sea environments [6,17,56–60]. As has been shown previously, most of them are common deep-sea bacteria, such as *Halomonas* and *Marinobacter* mixed with abundant Epsilonproteobacteria such as *Arcobacter*, *Sulfurimonas* and *Caminibacter*.

Epsilonproteobacteria are widespread at deep-sea hydrothermal vents, both as endo- and epibionts, and free-living [7,16,61]. Cultured members of mesophilic Epsilonproteobacteria have been found to exhibit heterotrophic and autotrophic oxidation of reduced sulfur, and the ability to use hydrogen as an electron donor [15], promoting their existence at hydrothermal vent environments. The ability of certain Gammaproteobacteria, such as the family SUP05, to oxidize sulfur has also been widely demonstrated [62,63]. The family SUP05 (Gammaproteobacteria) had a much higher sequence abundance around vent-influenced water, which is consistent with the possibly elevated concentration of hydrogen and sulfur from vent samples compared to the background samples (electronic supplementary material, figure S2). The presence of relatively abundant putative chemosynthetic organisms indicates that vent effluents directly sustain them. When hydrothermal fluids and seawater mix, either in the subsurface, above the seafloor or by advection across deposit walls, the fluids quickly reach characteristics of the ambient seawater, resulting in most marine hydrothermal vent habitats being circum-neutral. When plume water reaches neutrality to the characteristics of the ambient water there are fewer potential electron donors available for the microbial communities it hosts. This is evident from the diffuse vents and the less-hydrothermally affected samples, which have a community structure that is more similar to the background samples than the vent plumes (figure 6). However, the vent effluents could reach circum-neutrality more rapidly than the chemical oxidation of reduced compounds, leaving electron donors to be used by microbes despite a circum-neutral pH and a low signal from the redox potential sensors. It is evidently clear from the diffuse samples that hydrothermal fluids advecting through the seafloor have an impact on the microbial communities (figure 5) and consequently on fresh carbon being fixed in the deep oceans. It is widely known that the initial high temperatures from vents create the underlying chemical environment for microbial growth and thus influence the abundance of these microorganisms. Even at a lower temperature and likely lower influx of chemical species from the Earth’s mantel, the diffuse vents still compose a unique habitat from the background deep-sea water.

### 4.2. Vent biogeography

Weak biogeographic signals in microbial communities are usually explained by the hypothesis of microbial cosmopolitanism formulated by Baas Becking [64]. However, recent studies have explored the effects of dispersal limitation on microbial biogeography. The biogeography of the hydrothermal vent-associated microbial community has been studied before [5,14,23,25,65,66] and members of the genera presented in this study have been found globally at hydrothermal vent fields [7,26]. However, their relative abundance and heterogeneities between vent fields are poorly understood. It has recently been shown that genetic variation of the Epsilonproteobacteria *Sulfurimonas* are linked to geographical distance and not the geochemistry of a given vent environment [26]. Although, a hydrothermal vent environment will foster the growth of certain microorganisms, apparent from Dog’s Head and Longqi. Free-living microorganisms in the water column within hydrothermal plumes have only been studied on few occasions [4–7,67,68] and never at the vent fields in the ESR and SWIR. The two active vents, Dog’s Head and Longqi, clearly show a great similarity in their community structure (figure 6), again indicating environmental selection of certain organisms.

Recent exploration of hydrothermal vents has led to a biological classification of 11 provinces based on the biogeography of vent metazoan macro- and megafauna [8,69]. These provinces were based on multicellular organisms only, although microorganisms have been suggested to show unique patterns around vents [25,26]. It has also been suggested that arc and backarc hydrothermal systems may represent bacterial hotspots of diversity in the ocean [70], emphasizing the importance of including the complex communities of microorganisms from vents into biogeographic classification schemes. Community structures of this study clearly elucidate the larger differences between the vent and non-vent samples than the basin scale differences between the two vent sites. However, these two sites are also distinct from each other (PERMANOVA, *p*<0.01), likely because of differences in the environment. Future research should investigate the global biogeography of free-living microorganisms around hydrothermal vents comparing the microbial biogeography to that of metazoan vent communities [8,69,71,72]. It has been postulated that certain microorganisms might disperse with macroorganisms, thus, including microorganisms into these biogeographic regions might be more meaningful as a measure of habitat construct (i.e. community structure and connectivity between vent sites).

### 4.3. Dilution of microbial communities

Similar to Read *et al.* [73] for riverine systems, we propose that the observed pattern in microbial community composition is related to water residence time or current speed and to changing resource availability [38,74]. Firstly, hydrothermal plumes vary in their dispersion depending on the temperature of the plume origins, current speed and direction. As water transitions away from the plume, the microbial community becomes dispersed and eventually resource limited, and is unable to survive in the surrounding deep-sea water. This is supported by the decrease in abundance of known vent taxa with distance from plume (figure 5). The pattern is especially clear for the succession of the Epsilonproteobacteria composition with exponential decline with distance from plume, transitioning from high abundance closer to the vent to a lower abundance farther away (figure 7) [14,75]. The relationship between sequence abundance and cell abundance is not always straightforward and the problem of multiple copies of the 16S rRNA gene from the same cell on the evaluation of abundance has been raised [76]. However, with the sequencing depth achieved here, we can distinguish between the main bacterial players in and between the samples and locations and would argue that the abundance of Epsilonproteobacteria to vent-distance pattern is representative of the community composition, given the 100–300-fold increase of abundance between the plume and the background (figure 7).

The hydrothermal vent plume creates a dynamic environment with high-temperature fluids mixing with ambient seawater. This dynamic environment increases the amount of available resources and potential electron donors for growth and reproduction, which could lead to higher competition pressure and slightly higher taxa richness seen closer to the vent plumes (electronic supplementary material, figure S1). Sheik *et al.* [5] suggested that the persistent abundance of vent-associated microorganisms (e.g. SUP05 and Epsilonproteobacteria) in vent plumes suggests that either (i) they grow rapidly to keep pace with dilution by background seawater or (ii) they are entrained from surrounding water. Based on the nature of mesophilic Epsilonproteobacteria, it would require a rapid re-introduction rate of organisms to retain the abundance seen close to the plumes. Thus, a significant amount of the Epsilonproteobacteria in the diffuse flow and hydrothermal fluids are most likely originating from the seafloor discharged from the mixed fluids closer to the source at the seafloor resulting in the observed distance–decay relationship [5]. The Epsilonproteobacteria are so highly correlated with the hydrothermal plume that they could function as indicators of hydrothermal activity and as passive tracers of the vent fluid that originally contained a high abundance of these organisms. However, to properly consider the mechanisms of these communities the hydrodynamic fluxes would need to be taken into account, where a greater fluid flux, and distance from seafloor, have been suggested to increase entrainment of near-bottom seawater into the plume [5,77]. Samples collected using an remotely operated vehicle have shown that the early stages of the plume are substantially influenced by seafloor-derived microbes (Epsilonproteobacteria), and that these populations give way to seawater- and/or plume-adapted organisms (such as SUP05) as the plume rises and dilutes [5]. A clear pattern of SUP05 abundance increase with distance from vent was not observed in this study; however, SUP05 was most abundant around the plume and the diffuse flow, indicating some adaptation to hydrothermally derived electron donors. Although efforts were made to collect samples with maximum hydrothermal influence, samples collected with a CTD cannot substitute the precision of samples collected with remotely operated or human operated vehicles. However, the sequence abundance of Epsilonproteobacteria increased and Gammaproteobacteria decreased across the full range of observed Eh values, revealing a similar community turnover as has been seen previously [5].

The Epsilonproteobacteria dilution from the plume may be a good demonstration of the dispersion of relatively rare bacteria in the water column and a great example of the limitations of bacterial niche-specificity to their habitat, supporting the idea of environmental selection [64]. The cell abundance of microorganisms was higher in the diffuse flow compared with the vent plume indicating a higher sustaining influence of the diffuse flow than the core vent plume, possibly due to a lower level of high-temperature fluids being introduced to the seawater resulting in a more stable environment for proliferation compared with the vent plume, where water is continuously being forced away from the vent orifice. Our data suggest that hydrothermal vents and diffuse flow influence the abundance and community composition of microbial communities. These systems clearly sustain certain microorganisms in the surrounding environment, which influences the deep-sea carbon input and helps sustain the macroorganisms around the hydrothermal vents and the deep sea.

## Supplementary Material

Figure S1: This figure shows a boxplot of the OTU richnesses between all CTD casts collected on the East Scotia Ridge and the Southwest Indian Ridge.

## Supplementary Material

Figure S2:This barplot figure is of the sequence abundance of all the proteobacteria (A), and the SUP05 (B) from East Scotia Rdige hydrothermal vents, diffuse flow, and background samples. The y-axis is Eh, which represents a measure of how influenced the sample is by hydrothermal activity. The arrow represents the increase of hydrothermally influenced samples.

## Data accessibility

All sequencing and environmental data are available through Dryad: http://dx.doi.org/10.5061/dryad.qh767 [78].
