# Supplementary figures and images for: Cutting through the smoke: the diversity of microorganisms in deep-sea hydrothermal plumes
Source: R Soc Open Sci. 2017 Apr 12;4(4):160829. doi: 10.1098/rsos.160829 (PMC5414241; doi:10.1098/rsos.160829)

Observed richness

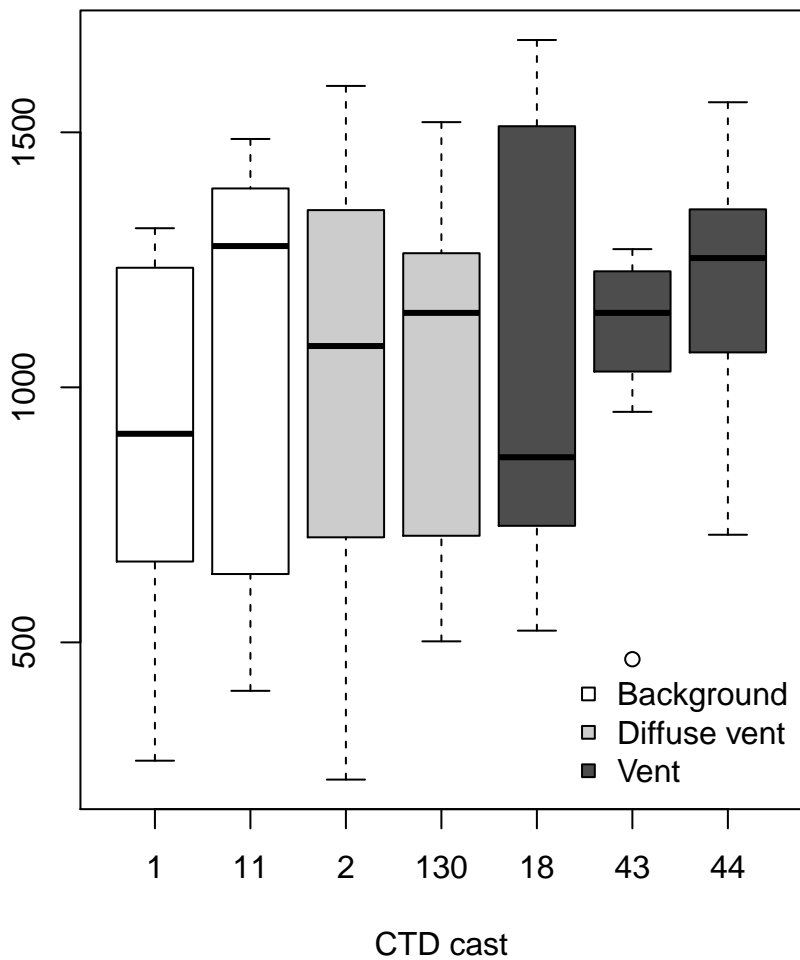

Supplement: Figure S1: This figure shows a boxplot of the OTU richnesses between all CTD casts collected on the East Scotia Ridge and the Southwest Indian Ridge. [file rsos160829supp1.pdf]

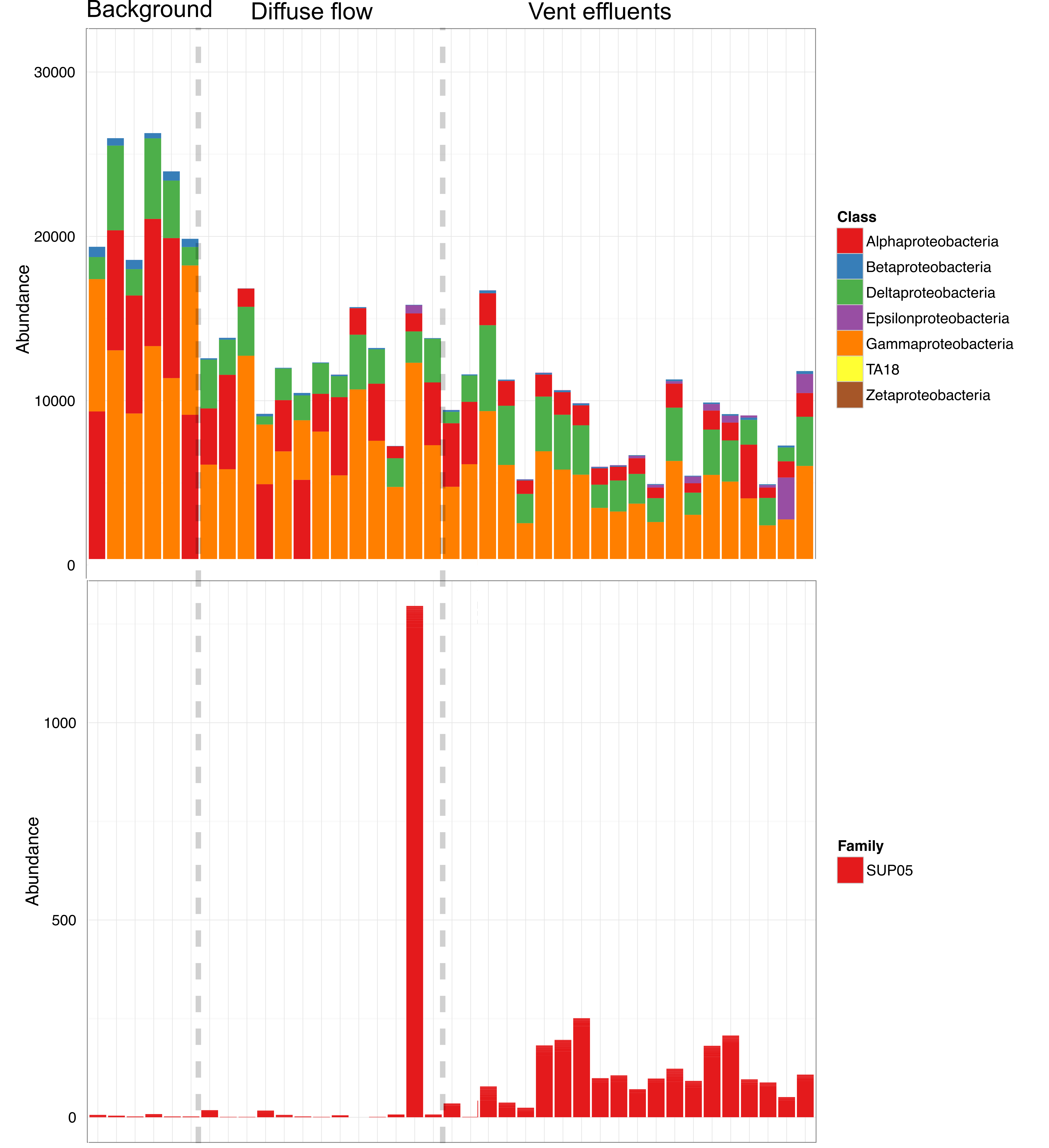

Supplement: Figure S2:This barplot figure is of the sequence abundance of all the proteobacteria (A), and the SUP05 (B) from East Scotia Rdige hydrothermal vents, diffuse flow, and background samples. The y-axis is Eh, which represents a measure of how influenced the sample is by hydrothermal activity. The arro [file rsos160829supp2.png]
